# Left ventricular fibrosis in arrhythmic mitral valve prolapse: quantification and comparison of semi-automated techniques assessed by cardiac magnetic resonance

**DOI:** 10.1007/s10554-023-03006-6

**Published:** 2023-12-23

**Authors:** Annagrazia Cecere, Alberto Cipriani, Manuel De Lazzari, Francesca Graziano, Giulia Brunetti, Giorgio De Conti, Raffaella Motta, Alberto Ravagnin, Giulia Lorenzoni, Dario Gregori, Cristina Basso, Francesco Tona, Yoo Jin Lee, Francesca Nesta Delling, Sabino Iliceto, Martina Perazzolo Marra

**Affiliations:** 1 Cardiology Unit, Department of Cardiac, Thoracic, Vascular Sciences and Public Health, University of Padua–Azienda Ospedaliera, Via Giustiniani, 2, 35128 Padua, Italy; 2 Radiology Unit, University of Padua–Azienda Ospedaliera, Padua, Italy; 3 Department of Medicine, University of Padua–Azienda Ospedaliera, Padua, Italy; 4 Department of Cardiac, Thoracic, Vascular Sciences, and Public Health, Unit of Biostatistics, Epidemiology and Public Health, University of Padova, Padova, Italy; 5 Cardiovascular Pathology Unit, University Hospital of Padua, Padua, Italy; 6 Clinical Radiology, Cardiac and Pulmonary Imaging, University of California, San Francisco, CA USA; 7 Department of Medicine (Cardiovascular Division), University of California San Francisco, San Francisco, CA USA

**Keywords:** Arrhythmic mitral valve prolapse, Late gadolinium enhancement, Myocardial fibrosis, Cardiac magnetic resonance

## Abstract

**Purpose:**

Left ventricular (LV) fibrosis has a key role in arrhythmogenesis in patients with mitral valve prolapse (MVP). Cardiac magnetic resonance identifies LV fibrosis by using late gadolinium enhancement (LGE) technique. LGE assessment and quantification in patients with MVP lacks of standardization protocols.

**Methods:**

66 MVP patients with normal systolic function and without significant regurgitation were enrolled. Semi-automated gray-scale thresholding techniques using full width at half maximum (FWHM) and 2, 3 and 5 standard deviation (SD) above the remote myocardium were used and compared with the visual assessment, considered as the gold standard.

**Results:**

LGE was identified in 41 MVP patients (62%) and quantified. The mean quantity of LGE visually assessed was 2.40 ± 1.07% or 1.40 ± 0.82 g. With FWHM, LGE resulted 3.56 ± 1.23% or 1.99 ± 1.13 g. Using thresholding, the mean LGE quantity was 9.2 ± 3.1% or 4.82 ± 2.28 g for 2-SD, 5.72 ± 1.75% or 3.06 ± 1.47 g for 3-SD and 2.36 ± 0.99% or 1.29 ± 0.79 g for 5-SD. The 5-SD measurement in percentage demonstrated a good correlation with LGE quantification visually assessed (2.40 ± 1.07 vs. 2.363 ± 0.9909, p = 0.543). When compared with the gold standard, the 5-SD threshold quantification, both in percentage and in grams, revealed the least intra-observer (respectively, ICC: 0.976 and 0.966) and inter-observer variability (respectively ICC: 0.948 and 0.935).

**Conclusion:**

The 5-SD gray-scale threshold technique in percentage revealed the best correlation with the visual assessment and an optimal reproducibility in MVP patient.

## Introduction

Mitral valve prolapse (MVP) is the most common degenerative valve disease and the primary cause of mitral regurgitation (MR) in the Western countries, affecting up to 3% in the general population [1]. Despite been considered as a benign condition, sudden cardiac death (SCD) cases have been reported and left ventricular (LV) fibrosis has been shown a frequent finding in patients with MVP and SCD [2, 3]. The pathogenesis of LV fibrosis in MVP is complex and not fully elucidated, but a role of MVP-induced myocardial stretch has been postulated [4, 5]. Cardiac magnetic resonance (CMR) provides a comprehensive assessment of LV fibrosis by the mean of the late gadolinium enhancement (LGE) technique [2]. In patients with MVP, LGE is a frequent finding, and is associated with mitral valve apparatus abnormalities, LV remodelling, MR grade, electrical instability, and cardiovascular events [6, 7]. However, to date, LGE assessment and quantification lacks of standardization protocols, and this may limit comparison of results among different studies and sites [8]. Multiple semi-automated methods for LGE quantification exist [9], including the use of signal intensity (SI) thresholds cut-off values of 2 to 6 standard deviations (SDs) above the remote non-enhanced myocardium or the use of half the maximal signal within the scar as the threshold (full-width at half maximum, FWHM) [9, 10], but to date there is not clear consensus regarding which technique is the most reliable and reproducible. Therefore, we evaluated the most reproducible technique for quantifying LGE and its clinical relevance in terms of arrhythmias in a large cohort of patients with MVP without valve regurgitation.

## Materials and methods

### Study populations

This retrospective study included 90 consecutive patients referred to the Cardiology Unit in Padua from September 2010 to December 2019 with echocardiographic diagnosis of “classical” MVP [2, 5] and who underwent CMR for presence of ventricular arrhythmias (VAs), previously detected (electrocardiogram, exercise stress test or 12-lead 24-hour Holter monitoring). To avoid inclusion of both non-classical MVP and fibroelastic deficiency, MVP was defined as > 5-mm thickening and > 2-mm displacement of one or both mitral leaflets in the left atrium, as viewed in the orientation of the LV outflow tract [4, 11].

Exclusion criteria were: moderate-to-severe MR, LV systolic function (LVEF) < 50%, tricuspid dysplasia or regurgitation, cardiomyopathies or congenital heart abnormalities, hemodynamic unstable conditions and contraindication to CMR.

The study was approved by the institutional review and all patients gave informed consent.

### Cardiac magnetic resonance imaging

#### Acquisition protocol

CMR was performed on a 1.5-T scanner (Magnetom Avanto, Siemens Healthineers, Erlangen, Germany), using a comprehensive dedicated protocol, as previously reported [2–4, 12].

All LGE images were acquired ten minutes after intravenous administration of contrast agent (gadobutrol, Gadovist; Bayer; 0.2 mmol/kg of body weight) in the same views of the cine images, covering the entire ventricles [12]. Inversion time were adjusted in order to neutralize the normal myocardium signal using a Look-Locker sequence. Images were repeated in two separate phase-encoding directions in order to exclude artefacts.

### Image analysis

**Fig. 1:**
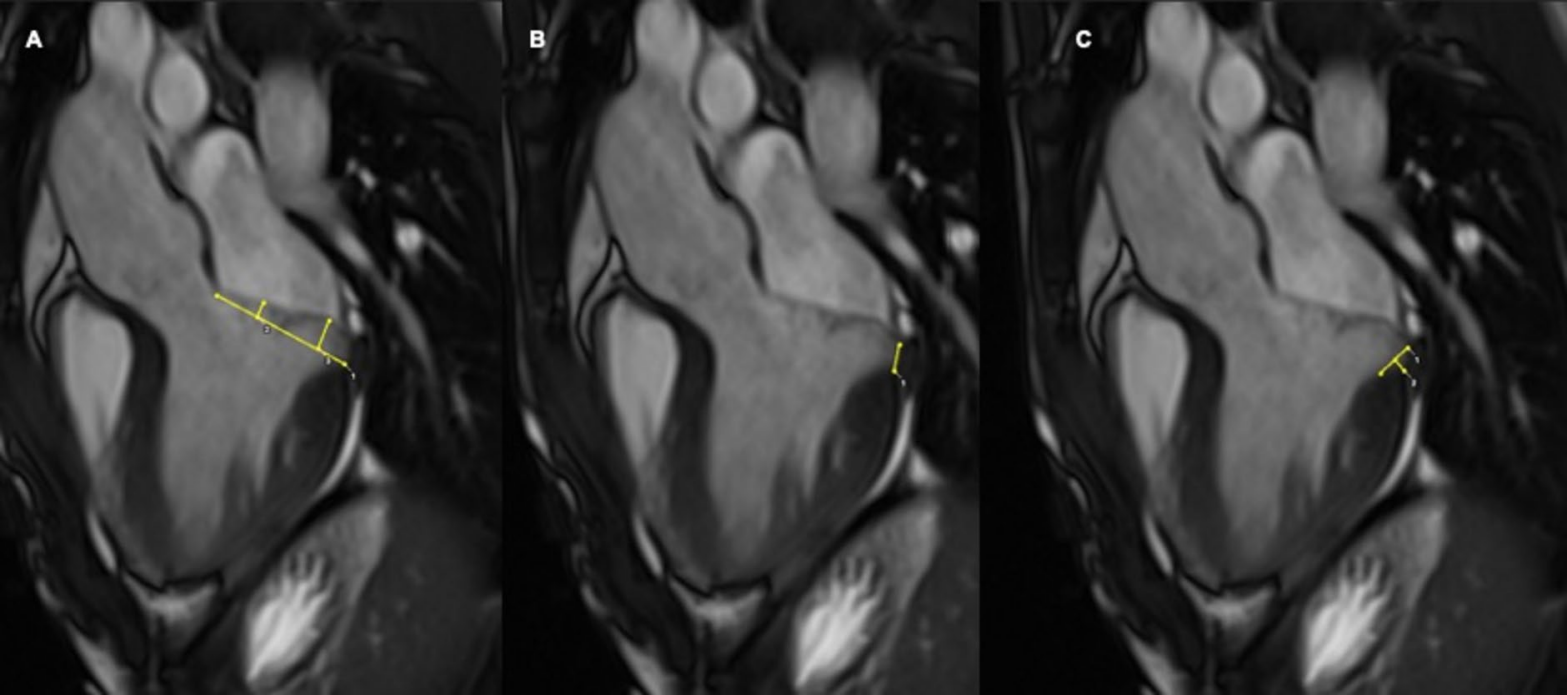
Cardiac magnetic resonance cine images in patient with mitral valve prolapse. **A** On 3-chamber, long-axis view, In end-systole, the prolapsed distance is measured as the maximum distance of the anterior and the posterior leaflet beyond the mitral annulus. **B** On the same end-systolic frame, MAD measurement is performed. MAD is measured from the left atrial wall -posterior MV leaflet junction to the top of the left ventricular infero-basal wall. **C** Curling measurement is provided in end-systole on 3-chamber long-axis view. A line between the top of the LV inferobasal wall and the LA wall-posterior MV leaflet junction is traced, and from this line, a perpendicular line to the lower limit of the mitral annulus

Morpho-functional analysis included biventricular volume and systolic function, prolapsed distance (measured as the maximum prolapsed distance during peak systole beyond the mitral annulus), basal and mid ventricular end-diastolic wall thickness and its ratio and variation of mitral annular diameter during end-systole and end-diastole [3, 13–15]. Finally, presence of mitral annular disjunction (MAD) and curling were assessed, as previously proposed [3] (Fig. 1). MAD has been described as a separation between left atrial wall at the level of MV junction and the LV free wall [3]. Conversely, curling is an abnormal anomalous systolic movement of the LV inferolateral wall, secondary to MAD. MAD and curling has been recognized as typical findings in MVP patients, as the morpho-functional alterations that could promote, time by time, the replacement type-fibrosis in the basal and mid inferolateral wall.

On dedicated LGE images, the LV endocardial and epicardial borders were manually traced for determination of myocardial mass, excluding papillary muscles and intertrabecular blood pool from the total myocardial mass. In order to exclude false positive images, LGE was considered present only if visible in two orthogonal views. Myocardial LGE quantification were assessed by two cardiologists (by A.C. and A.C.) with more than 3 years of experience in CMR imaging.

Normal myocardium was defined as a region of myocardium without any apparent bright appearance suggestive of scar. Visually LGE assessment was performed with manual planimetry of all enhanced pixels on the short-axis post-contrast images [16]. For comparison, a semi-automated gray-scale thresholding technique was performed using the 50% of the SI of the hyper-enhanced area (for the FWHM method) and a 2-SD, 3-SD and 5-SD above the mean SI for the normal myocardium [10]. For the FWHM threshold, a region of interest (ROI) in the maximum hyperintense myocardium was traced and used to define maximal signal [17]. Conversely, the mean SI and SD were evaluated by drawing a ROI in a portion of normal myocardium on three consecutive short-axis images (a sample of at least 100 pixels per ROI). The mean SI and SDs were consequently calculated across the three short-axis images as an average mean SI and SD.

LGE quantification was expressed in grams and as percentage of the total LV myocardial mass (M_LGE_/M_M_) × 100 with LGE mass (M_LGE_) and myocardial mass (M_M_) expressed in grams [16] .

Inter-observer agreement was performed by an expert CMR cardiologist (by M.P.M.), blinded to previous analysis, in 16 studies. Intra-observer agreement was assessed for all cases two months after the first analysis.

All CMR images were analysed using a dedicated postprocessing software (CVI^42^ version 5.11.4, Circle Cardiovascular Imaging Inc, Calgary, Canada).

### Statistical analysis

Descriptive statistics were reported as absolute frequencies (percentage) for categorical variables; median with 25 to 75 percentiles and mean (± SDs) for continuous variables. Comparison by groups were made using the Chi-squared test (or the Fisher exact test) and Student’s t test (or Mann–Whitney U test), as appropriate. P ≤ 0.05 was considered statistically significant. Paired Wilcoxon test was used to compare distributions of LGE measurements collected with visual assessment and other semi-quantitative methods. Bland-Altman plots were implemented in order to visually investigate the agreement between visual assessment and the different LGE semi-automated quantification methods.

Intra-class correlation coefficient (ICC) was calculated as absolute agreement to measure intra-operator and inter-operator agreement for each method. An ICC ≥ 0.9 was considered as an optimal concordance.

The analysis was performed using R software [18] with packages BlandAltmanLh [19] and irr [20].

## Results

From the initial cohort, 24 patients were excluded for LV systolic dysfunction (n = 9) and significant MR (n = 15). Therefore, the final cohort was constituted by 66 MVP arrhythmic patients with normal systolic function and absent/trivial MR. Clinical characteristics of the entire enrolled population were summarized in Table 1.

**Table 1:** Clinical characteristics and cardiac magnetic resonance findings in MVP patients without and with LGE

	MVP patients without LGE (25 pts)	MVP patients with LGE (41 pts)	p
Clinical characteristics			
Age (median)	47 (29–54)	43 (40-55)	0.937
Female, n (%)	15 (60.0%)	32 (78.0%)	0.099
Symptoms, n (%)	16 (64.0%)	24 (58.5%)	0.430
Dyspnoea, n (%)	0 (0%)	3 (7.3%)	0.233
Chest pain, n (%)	1 (4.0%)	4 (9.8%)	0.367
Ventricular arrhythmias, n (%)	5 (20.0%)	21 (51.2%)	**0.011**
Syncope, n (%)	3 (12.0%)	4 (9.8%)	0.538
Cardiac arrest, n (%)	1 (4.0%)	1 (2.4%)	0.618
ECG pattern			
Negative T waves, n (%)	2 (8.0%)	5 (12.2%)	0.462
Negative inferior T waves, n (%)	2 (8.0%)	4 (9.8%)	0.590
Negative lateral T waves, n (%)	0 (0.0%)	1 (2.4%)	0.621
Negative V1–V3 T waves, n (%)	1 (4.0%)	0 (0.0%)	0.379
CMR morpho-functional findings			
LV EDV, ml/m^2^	90.0 (71.0 – 105.0)	93.0 (80.0 – 104.0)	0.625
LV ESV, ml/m^2^	31.0 (23.0 – 39.0)	37.0 (31.0 – 44.0)	**0.038**
LV EF, %	65.0 (61.0 – 68.0)	58.0 (56.0 – 61.0)	**< 0.001**
LV mass, g/m^2^	53.0 (48.0 – 60.0)	53.0 (45.0 – 64.0)	0.741
RV EDV, ml/m^2^	83.0 (64.0 – 96.0)	79.0 871.0 – 92.0)	0.984
RV ESV, ml/m^2^	29.0 (20.0 – 41.0)	33.0 (22.0 – 37.0)	0.587
RV EF, %	65.0 (60.0 – 70.0)	61.0 (57.0 – 69.0)	0.054
MVP leaflet involvement			
Anterior, n (%)	1 (4.0%)	1 (2.4%)	0.618
Posterior, n (%)	13 (52.0%)	7 (17.1%)	**0.003**
Bileaflet, n (%)	11 (44.0%)	33 (80.5%)	**0.003**
Prolapse distance, mm			
Anterior leaflet, mm	0.2 (0.0 – 3.0)	3.0 (1.0 - 5.6)	**0.002**
Posterior leaflet, mm	3.6 (2.1 – 5.0)	7.0 (4.6 – 10.0)	**< 0.001**
LV lateral wall thickness, mm			
Basal segment, mm	8.2 (7.0 – 10.0)	8.8 (7.0 – 11.0)	0.615
Mid segment, mm	4.5 (4.0 – 5.6)	4.6 (4.0 – 5.0)	0.665
Ratio LV basal/mid wall thickness	1.7 (1.5 – 1.9)	1.8 (1.7 – 2.0)	0.056
MVP-related morpho-functional alterations			
Systolic-diastolic variation mitral annular diameter, mm	8.0 (6.0 – 10.0)	11. 0 (7.0 – 15.0)	**0.011**
MAD, n (%)	9 (36%)	35 (85.4%)	**< 0.001**
MAD, mm	0.2 (0.0 – 4.6)	6.0 (4.8 – 8.0)	**< 0.001**
Curling, n (%)	10 (40.0%)	34 (83.0%)	**< 0.001**
Curling, mm	0.2 (0.0 – 2.9)	4.0 (3.0 – 5.0)	**< 0.001**

Significant differences are in bold (when P ≤ 0.05, as described in the Statistical analysis)

*MVP* mitral valve prolapse, *LGE* late gadolinium enhancement, *LV EDV* left ventricular end-diastolic volume, *LV ESV* left ventricular end-systolic volume, *LV EF* left ventricular ejection fraction, *RV EDV* right ventricular end-diastolic volume, *RV ESV* right ventricular end-systolic volume, *RV EF* right ventricular ejection fraction, *MAD* mitral annular disjunction

### LGE measurements comparison

**Fig. 2:**
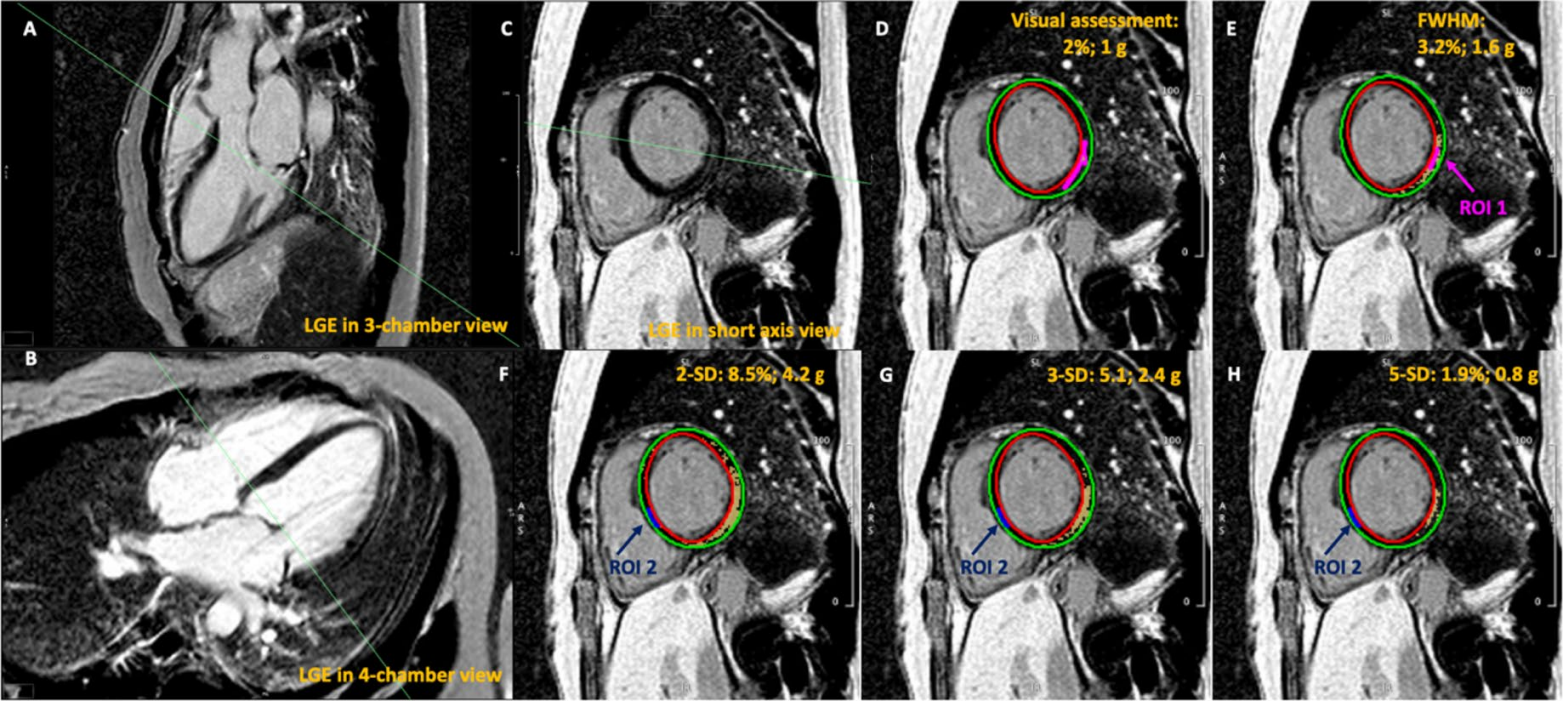
Late gadolinium enhancement semi-quantitative quantification techniques assessed by cardiac magnetic resonance. Typical intramural left ventricular (LV) inferobasal wall late gadolinium enhancement (LGE) in arrhythmic mitral valve prolapse (MVP) patients in long-axis (respectively,  **A** and **B**) and short-axis view (**C**), before planimetry and quantification were performed. Identical LV short-axis contrast enhanced images (**D**–**H**) revealed LGE, in percentage and grams, depicted at visual assessment (**D**), at full width at half maximum (FWHM), drawing a region of interest (ROI) in the maximum hyperintense myocardium (**E** ROI 1, purple arrow), and with different gray-scale thresholding techniques at 2, 3 and 5 SDs, above mean signal intensity of normal remote myocardium (**F**–**H**, ROI 2, blue arrow). Left ventricle endocardium and epicardium are respectively delineated in red and green. Yellow areas represent areas of delayed enhancement in different LGE semi-automated quantification methods

**Fig. 3:**
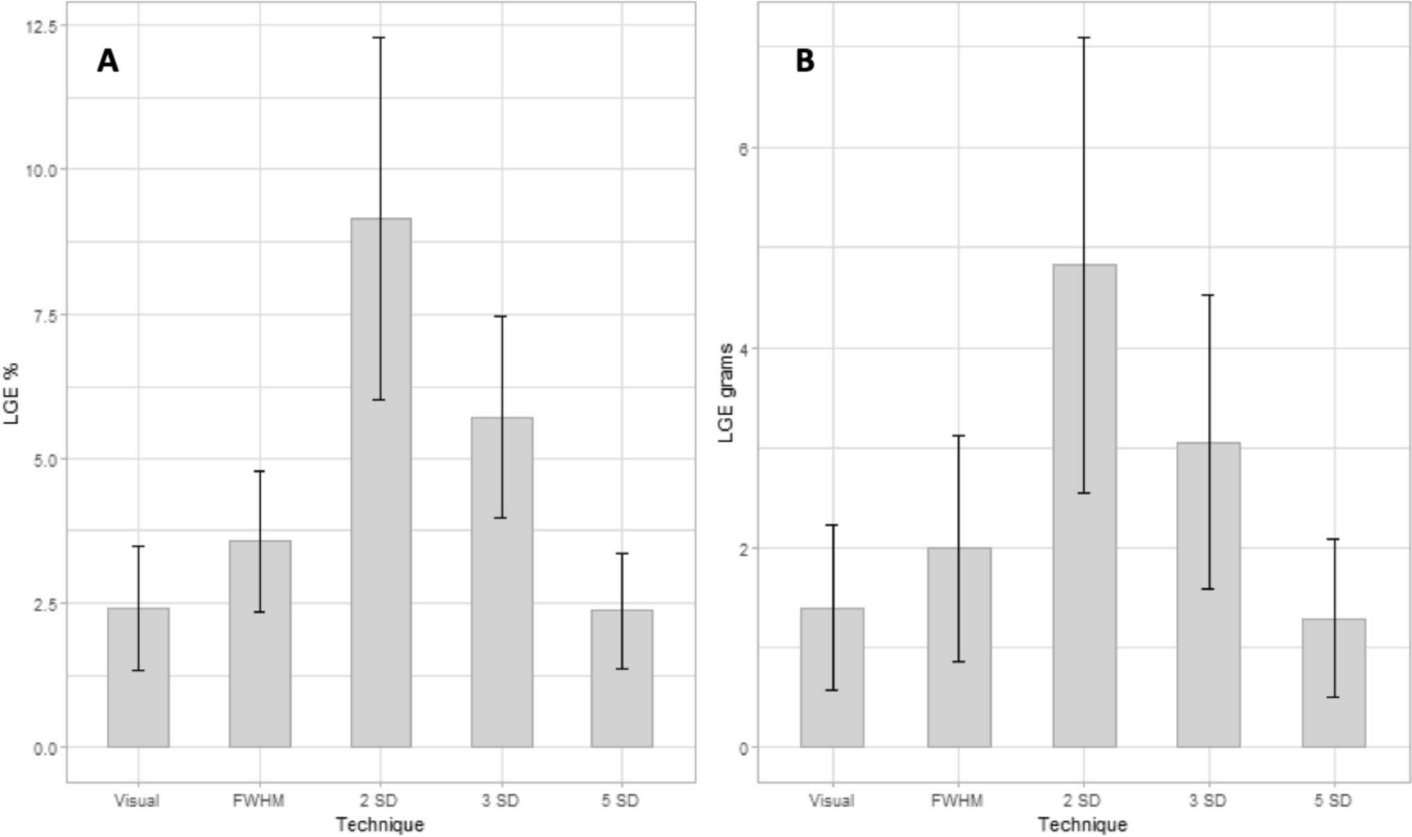
Difference in LGE (percentage and grams) between semi-automated quantification methods in MVP patients with LGE. The graphs illustrate volumes of late gadolinium enhancement (LGE) in percentage (**A**) and grams (**B**), measured as mean ± standard deviation, assessed by using visual assessment, full width at half maximum (FWHM) and different gray-scale thresholding techniques (2, 3 and 5 SDs above mean signal intensity for the normal myocardium). Comparing the different semi-quantitative LGE quantification methods, the greatest amount of LGE was measured with 2-SD method and the lowest with the 5-SD one

LGE was identified in 41 arrhythmic MVP patients (62%) and consequently quantified (Fig. 2). LGE extent quantification are summarized are summarized in Table 2. The mean extent of LGE visually assessed was 2.40 ± 1.07% (range, 1.50–3.00%) or 1.40 ± 0.82 g (range, 0.80–1.90). With FWHM, LGE resulted 3.56 ± 1.23% (range, 2.90–4.20%) or 1.99 ± 1.13 g (range, 1.29–2.29). Using different thresholding, the mean LGE amount was 9.2 ± 3.1% (range, 6.8–10.9%) or 4.82 ± 2.28 g (range, 3.18–6.0 g) for 2-SD, 5.72 ± 1.75% (range, 4.60–7.0%) or 3.06 ± 1.47 g (range, 2.12 ± 3.70 g) for 3-SD and 2.36 ± 0.99% (range, 1.60–3.10%) or 1.29 ± 0.79 g (range, 0.77–1.50 g) for 5-SD. As evidenced in Fig. 3, the greatest amount of LGE was measured with 2-SD method and the lowest with the 5-SD one.

**Table 2:** LGE measurements with different semi-quantitative different methods

	MVP patients with LGE (41 pts)	
	Mean	Median
LGE quantification (%)		
Visual assessment	2.40 ± 1.07	2.30 (1.50–3.00)
FWHM	3.56 ± 1.23	3.40 (2.90–4.20)
2-SD	9.2 ± 3.1	9.0 (6.8–10.9)
3-SD	5.72 ± 1.75	5.20 (4.60–7.00)
5-SD	2.36 ± 0.99	2.10 (1.60–3.10)
LGE quantification (g)		
Visual assessment	1.40 ± 0.82	1.20 (0.80–1.90)
FWHM	1.99 ± 1.13	1.86 (1.29–2.29)
2-SD	4.82 ± 2.28	4.41 (3.18–6.00)
3-SD	3.06 ± 1.47	2.55 (2.12–3.70)
5-SD	1.29 ± 0.79	1.17 (0.77–1.50)

*MVP* mitral valve prolapse, *LGE* late gadolinium enhancement, *FWHW* full width at half maximum, *SD* standard deviation

LGE localized on papillary muscles was only visually assessed, according to previous studies [21, 22].

All semi-quantitative methods for LGE quantification demonstrated a good intra and inter-observer agreement (Table 3) [23]. However, the 5-SD threshold quantification, both in percentage and in grams, revealed the least intra-observer variability (respectively, ICC: 0.976 and ICC: 0.966) and the least inter-observer variability (respectively ICC: 0.948 and ICC: 0.935) when compared with visual assessment.

**Table 3:** Intra and inter-observer agreement for different LGE semi-quantitative quantification techniques

	Intra-observer agreement	Inter-observer agreement
LGE quantification (%)	ICC	
Visual assessment	0.997	0.991
FWHM	0.927	0.913
2-SD	0.89	0.872
3-SD	0.955	0.911
5-SD	0.976	0.948
LGE quantification (g)	ICC	
Visual assessment	0.996	0.985
FWHM	0.704	0.812
2-SD	0.856	0.82
3-SD	0.929	0.846
5-SD	0.966	0.935

*ICC* intraclass correlation coefficient, *FWHW* full width at half maximum

### CMR morpho-functional findings in arrhythmic MVP patients

Compared with those without, MVP patients with LGE had a greater LV end-systolic volume (37 vs. 31 ml/m^2^, p: 0.038, Table 1) and a lower LVEF (58 vs. 65%, p < 0.001), although within the normal limits, when compared to those without LGE. Bileaflet MVP was more frequent in MVP patients with LGE (33 vs. 11, p = 0.003); conversely, MVP patients without LV fibrosis demonstrated commonly an isolated posterior leaflet prolapse (13 vs. 7, p = 0.003) when compared with LGE positive cases. MVP patients with LGE revealed an increased systolic bileaflet atrial excursion (respectively, anterior leaflet: 3 vs. 0.2 mm, p = 0.002; posterior leaflet: 7 vs. 3.6 mm, p = < 0.001) and a greater systo-diastolic variation of mitral annular diameter (11 vs. 8 mm, p = 0.011), in comparison with MVP patients without LGE.

In addition, a greater LV mechanical stress in the LV myocardium, expressed by severe MAD (6 vs. 0.2 mm, both p < 0.001) and curling (4 vs. 0.2 mm, both p < 0.001), were reported in MVP patients with LGE when compared with those without fibrosis.

### Arrhythmic clinical impact of LGE measurement

No clinical differences between arrhythmic MVP patients without (n = 25) and with LGE (n = 41) were identified, except for VA (p = 0.011). ECG pattern demonstrated to be similar between the two groups.

**Fig. 4:**
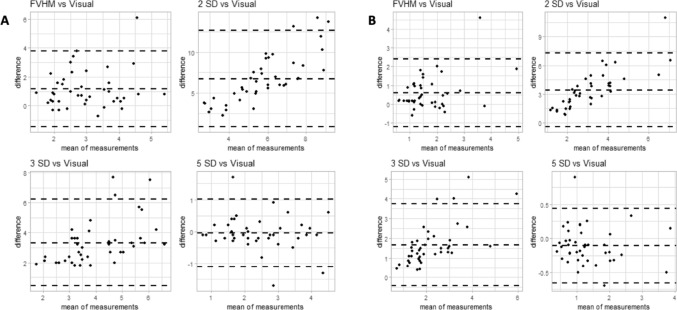
Bland–Altman plots in mitral valve prolapse patients with LGE. Bland–Altman plots show agreement between LGE measurements in percentage and grams (respectively figure **A**, **B**), made by visual assessment, full-width at half maximum (FWHM) and different gray-scale thresholding measurements (2, 3 and 5 SDs above mean signal intensity for the normal myocardium). LGE quantification in percentage made with 5-SD demonstrates the best correlation with LGE evaluated with visual assessment. On each plot, the center line represents overall bias, and top and bottom lines represents 95% agreement between methods

In arrhythmic MVP patients, only 5-SD measurements in percentage demonstrated a good correlation with LGE quantification visually assessed (2.363 ± 0.9909 vs. 2.402 ± 1.075, p = 0.543, Table 3, Fig. 4). Conversely, all other LGE measurements, both in percentage and in grams, showed a statistically significant difference and a great difference with LGE visually assessed (Tables 4 and 5).

**Table 4:** Comparison between distributions of LGE measurements collected with visual assessment and other semi-quantitative methods, using paired Wilcoxon test

	MVP patients with LGE (41 pts)	p
LGE quantification (%)		
Visual assessment	2.402 ± 1.075	
FWHM	3.559 ± 1.23	< 0.001
2-SD	9.159 ± 3.125	< 0.001
3-SD	5.72 ± 1.748	< 0.001
5-SD	2.363 ± 0.9909	**0.543**
LGE quantification (g)		
Visual assessment	1.395 ± 0.8207	
FWHM	1.995 ± 1.132	< 0.001
2-SD	4.822 ± 2.275	< 0.001
3-SD	3.06 ± 1.474	< 0.001
5-SD	1.286 ± 0.7884	0.005

In this table, only the 5-SD measurements (%) presents a good correlation with LGE quantification visually assessed (in bold). In all the other measurements, both in percentage and in grams, a correlation with LGE quantification visually assessed wasn’t identified

*MVP* mitral valve prolapse, *LGE* late gadolinium enhancement, *FWHW* full width at half maximum, *SD* standard deviation

**Table 5:** Correlations between visual assessment and different semi-automated LGE quantification techniques assessed by cardiac magnetic resonance

	Bias	Lower	Upper
LGE quantification (%)			
FWHM	1.15	− 1.46	3.77
2-SD	6.75	0.87	12.64
3-SD	3.32	0.43	6.21
5-SD	− 0.04	− 1.09	1.02
LGE quantification (g)			
FWHM	0.60	− 1.21	2.40
2-SD	3.43	− 0.43	7.28
3-SD	1.67	− 0.42	3.76
5-SD	− 0.11	− 0.66	0.44

## Discussion

In our study, semiautomated LGE CMR semiautomated 5-SD gray-scale threshold technique in percentage revealed the best correlation with the visual assessment analysis, and an optimal reproducibility in patients with MVP. We demonstrated for the first time that semi-quantitative the 5-SD gray-scale technique is a reliable and reproducible method to accurately quantify LGE in MVP patients, thus providing a standardized approach to arrhythmic risk stratification. Finally, differentiating MVP patients on the basis of presence or absence of ventricular arrhythmias, in arrhythmic MVP patients, only 5-SD measurements in percentage demonstrated a good correlation with LGE quantification visually assessed.

### Quantification of LGE in MVP

CMR has been shown to accurately recognize myocardial LV macroscopic fibrosis with good correlation with pathology, both for ischemic and non-ischemic diseases [24–26]. Due to its excellent spatial resolution and high contrast-to-noise ratio, CMR clearly identifies LV macroscopic fibrosis as an evident bright high signal intensity area, visibly different from the dark normal myocardium, on T1 inversion recovery post-contrast images. In addition, on the basis of the LGE localization, CMR could differentiate ischemic cardiomyopathy, characterized by subendocardial to transmural LGE distribution, from the non-ischemic one, presenting with intramural or subepicardial LGE localization [25–27].

Overall, fibrosis is common in MVP (28–37%), [6, 7] usually located close to the annulus in the basal left ventricular wall, moreover only LGE within the mitral apparatus (papillary muscles and peri-annular region) has a clear pathophysiological association with arrhythmia. In fact, myocardial LV macroscopic fibrosis has a pivotal role in ventricular arrhythmogenesis, impacting on the prognosis of many cardiomyopathies [28–31].

In previous studies, different semi-automated techniques for LGE have been evaluated in various clinical settings, leading to variable amounts of LGE. Therefore, these semi-automated techniques may not be interchangeable, but should rather be tailored to LGE pattern and distribution and can be used as a validation tool.

Different studies showed demonstrated LGE threshold or FWHM techniques to best correlate to gross examination of LGE in various pathology. Ischemic LGE is characterized by a dense and homogeneous bright infarct core surrounded by a peri-infarct border zone, responsible for a high contrast area compared with the normal myocardium. Bondarenko et al. demonstrated a good correlation between LGE quantification with visual assessment and the 5-SD grey-scale threshold technique in patients with chronic ischemic heart disease [32]. According to this study, Vermes et al. confirmed that the use of low threshold considerably overestimated the real infarct size when compared to visual assessment [33].

Another paradigmatic cardiac disease in which LGE quantification is crucial for arrhythmic stratification, as supported by recent Guidelines [34], is represented by HCM in which has been demonstrated that the 6-SD gray-scale threshold technique presents the closest approximation with visual assessment and the best reproducibility [9, 16].

Although the pivotal role of LGE in ventricular arrhythmogenesis and prognosis in MVP patients has been widely demonstrated [35], a standardized method of LGE quantification has yet to be developed [2, 3]. Moreover, the semi-quantitative methods evaluated in other cardiomyopathies cannot be applied to arrhythmic MVP patients because the LGE pattern distribution and localization in this clinical setting are peculiar. The pathogenesis of myocardial fibrosis in arrhythmic MVP is complex and not really completely clarified. In our population, MVP patients with LGE demonstrated a greater mitral prolapse distance in the left atrium, a larger systo-diastolic variation of the mitral annular diameter, as well as a more severe MAD and curling, in comparison with patients without fibrosis (Table 1). Accordingly, arrhythmic MVP patients presented a mid-wall or subepicardial LGE stria in the basal or mid (in the site of papillary muscles attachment) inferolateral wall [3]. Our previous study [2] revealed that fibrosis in MVP patients is due to a replacement-type, but also to an interstitial fibrosis, leading to a different concentration of LGE. As well as for HCM in which LGE isn’t a synonymous of fibrosis, a specific LGE quantification method should be considered for MVP patients. Supporting this hypothesis, in our population the LGE amount, both in percentage and in grams, resulted slighter than in MI, HCM and suspected myocarditis evaluated in previous studies [10, 16, 17].

In accordance to prior studies, we measured the greatest amount of LGE with the 2-SD method and the lowest one with the 5-SD method, both in percentage and grams [9, 17] (Table 2; Fig. 3). Therefore, we used visual assessment, representing the human thresholding in bright signal identification, as a comparison method with the different semi-automated techniques (Table 4). In comparison with visual assessment, only the 5-SD quantification method in percentage didn’t significantly differ from the used gold standard (p = 0.543), reporting the least difference with the visual assessment (bias−0.04, Table 5). Intriguingly, the 5-SD quantification in grams showed a not neglectable difference with the visual assessment quantification (p = 0.005; bias−0.11, Table 5). This result could be explained by two phenomena. First, we identified a smaller LGE amount in MVP patients than other cardiomyopathies, that could minimize the differences between the used methods in percentage and grams. Secondly, the enrolled population is highly selected representing the true “malignant MVP” without the bias due to hemodynamic impairment since those with valve regurgitation were excluded. In fact, we excluded from the study all the MVP patients with other possible causes of LV fibrosis (such as moderate-to-severe MR or LV systolic dysfunction), different from the replacement-type, in order to quantify the real amount of LGE in MVP. Similarly, the other CMR semi-automated quantification methods revealed a significant difference with the visual assessment (p < 0.001).

In addition, we noticed that the use of a low gray-scale thresholding was associated with a great difference in the fibrosis amount compared with the visual assessment, responsible for a noteworthy overestimation of LGE (Fig. 4; Table 5), as previously reported in HCM patients [9, 16].

In comparison to the low gray-scale thresholding methods (the 2-SD and the 3-SD both in percentage and in grams), in our study FWHM demonstrated the least difference with the visual assessment (in comparison with visual assessment, respectively bias in percentage and in grams, 1.15 and 0.60, Table 5). However, despite it was more accurate than the low gray-scale thresholding methods, FWHM tended to overestimate the LGE amount when compared with the 5-SD (respectively, bias in percentage 1.15 vs. − 0.04 and in grams 0.60 vs. − 0.11, Table 5). This result seems to be in contrast with Flett AS et al. that demonstrated that FWHM technique represented the most accurate and reproducible LGE quantification method, regardless the disease [10]. In our case, this difference could be probably due to the different LGE pattern observed in MVP patients in comparison to MI and HCM included in the study of Flett AS et al. [10].

Intra and inter-operator agreement demonstrated a good reproducibility (in all used methods, ICC > 0.8). In particular, the visual assessment presented the best reproducibility, both in percentage and in grams. After the used gold standard, confirming the previous results, the 5-SD revealed an optimal concordance, both in percentage and in grams.

Nevertheless, due to the role of fibrosis in the natural history of arrhythmic MVP, prospective studies are necessary to evaluate the possible LGE remodeling in this setting.

### LGE measurements and arrhythmic MVP

Until about ten years ago, the risk of SCD was overt for MVP with severe regurgitation, only subsequently the extensive use of CMR a definite “malignant MVP” phenotype (beyond the valve incompetence), previously seen only by autoptic studies, has been recognized.

Morpho-functional anatomy of mitral valve, in term of severe myxomatous degeneration, MAD, replacement-type fibrosis, appears to play a crucial role in arrhythmic risk. This clinic-instrumental profile recognizes the LGE on CMR as a gatekeeper for arrhythmic risk stratification as highlighted by the recent EHRA Expert Consensus Statement [34]. Despite the increasing number of papers confirming the presence of LGE in MVP there is no standardization in the postprocessing of methods of delineating LGE, limiting comparison of results among different studies and sites [2, 3, 5, 6, 8, 35–37]. The postprocessing for LGE quantification has been standardized for several and different cardiac diseases [38] in order to provide not only a standardization for clinical purpose, but also for the research aims.

In the subset of malignant MVP data are limited to small study population, sometimes including also different grades of valve regurgitation, and multiple different methods of delineating LGE extent and defining the presence and extent of MAD further increase data heterogeneity [8]. Our study represents the application of different quantitative CMR tools for LGE assessment in a specific MVP population. The results indicate that the semiautomated 5-SD gray-scale threshold technique in percentage represents the more suitable methods in this setting, especially in those with ventricular arrhythmias. The question regarding the role of LGE in arrhythmogenesis in MVP, if it matters more the presence/absence or the total amount, remain to be elucidate, after the standardization of postprocessing protocol.

### Study limitations

This study is single center study with a selected number of patients, that may have limited the statistical analysis. Further multicenter studies, enrolling a higher number of MVP with and without valve regurgitation are needed, also taking into consideration the spatial resolution as a potential limit of the CMR methods. In accordance with literature and the postprocessing software, only myocardial LV LGE was quantified with semiautomated techniques; conversely, LGE localized on papillary muscles was only visually assessed [21, 22]. Finally, in the absence of gold standard semi-quantitative CMR method for LGE quantification, we used the visual assessment, accordingly to previous studies [10, 16].

## Conclusions

The presence of LGE is crucial for arrhythmic risk stratification in MVP but the lack of a standardization postprocessing protocol limits comparison of results among different studies and sites. In the light of these observations, we quantified LV fibrosis and compared different CMR semi-automated LGE quantification methods demonstrating that the semiautomated 5-SD gray-scale threshold technique in percentage revealed the best correlation with the visual assessment analysis and an optimal intra and inter-operator reproducibility, allowing an accurate LGE quantification in a population of arrhythmic MVP patients.
